# Outcomes of endoscopic chemo- and laser-cauterizations and open fistulectomy for pyriform sinus fistula

**DOI:** 10.1186/s40463-021-00537-7

**Published:** 2021-08-12

**Authors:** Hiroo Masuoka, Akira Miyauchi, Takahiro Sasaki, Tsutomu Sano, Akihiro Miya

**Affiliations:** 1 Department of Surgery, Kuma Hospital, 8-2-35 Shimoyamate-dori, Chuo-Ku, Kobe, Hyogo 650-0011 Japan; 2 Department of Head and Neck Surgery, Kuma Hospital, Kobe, Japan

**Keywords:** Pyriform sinus fistula, Laser-cauterization, Chemo-cauterization, Fistulectomy, Endoscopy

## Abstract

**Background:**

Acute suppurative thyroiditis through the congenital pyriform sinus fistula (PSF) often recurs if the fistula is not resected. Although endoscopic chemo-cauterization (ECC) to obliterate the orifice of the fistula is less invasive than open fistulectomy, it may require repeated treatments. We recently adopted an endoscopic diode laser-cauterization (ELC) system with the intention of improving treatment outcomes in PSF. Here, we describe ELC and compare the outcomes of these three modalities.

**Methods:**

We evaluated 83 patients with PSF who underwent treatment between 2007 and 2018 at Kuma Hospital, a tertiary thyroid treatment hospital. ECC and ELC were implemented in 2007 and 2015, respectively. Patients who were ineligible for the endoscopic procedures underwent open fistulectomy. Barium swallow studies and computed tomography scan under a trumpet maneuver were performed after treatment to evaluate obliteration or removal of the fistula.

**Results:**

In total, 70 of the 81 (86%) patients who underwent barium swallow studies after the first treatment achieved obliteration or removal of the fistula. The success rates for open fistulectomy, ECC, and ELC were 100% (9/9), 83% (49/59), and 100% (13/13), respectively. ECC and ELC had significantly shorter operative times and lower blood loss than open fistulectomy. Insufficient opening of the mouth was the major reason for converting endoscopic procedures to open fistulectomy.

**Conclusions:**

ELC may yield superior outcomes and is therefore the optimal treatment modality for PSF. However, it is still associated with certain limitations. Thus, treatment selection remains dependent on the shape and size of the PSF and the mouth opening of the individual patient.

**Graphical Abstract:**

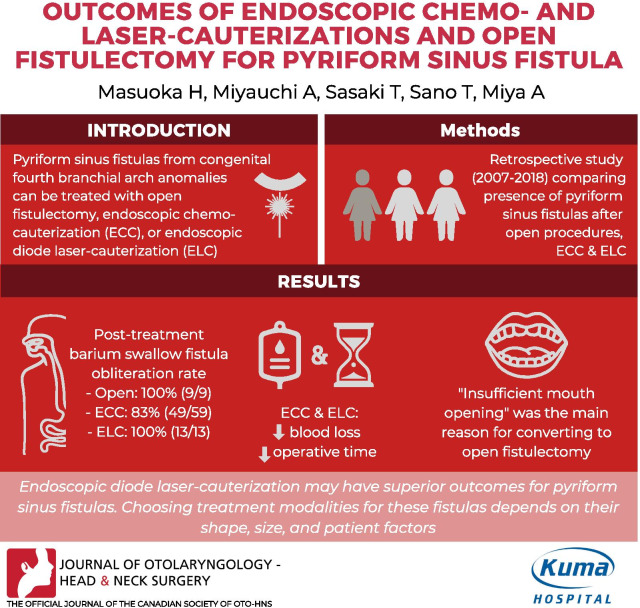

## Introduction

**Fig. 1:**
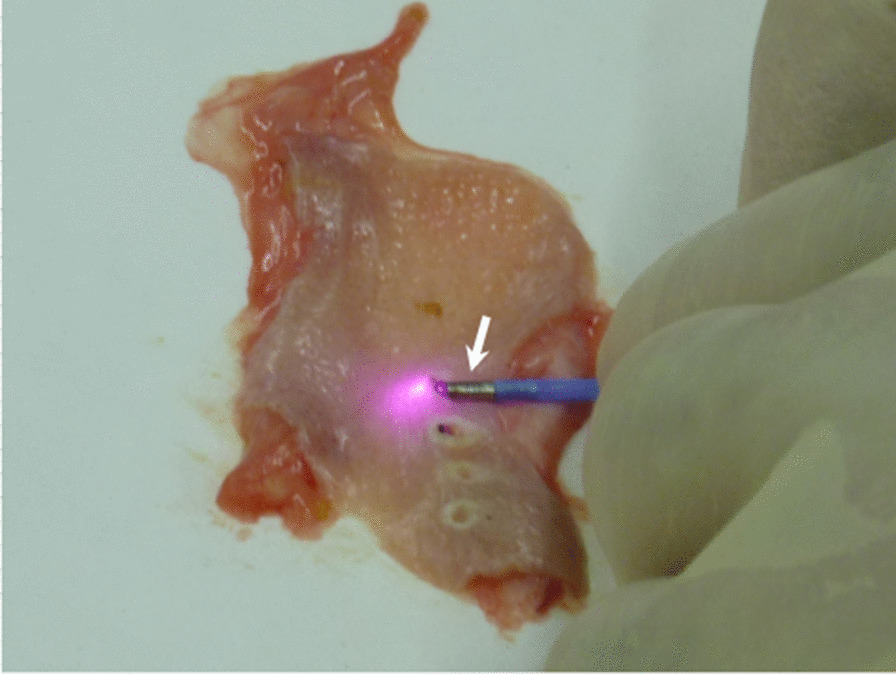
Image of the pre-clinical experiment using raw chicken esophagus. To test the amount of the heat sufficient to cauterize only its mucosal layer without heat denaturing its muscle layer. A Diode Laser System ADL-20 generated 810-nm wavelength laser during the active mode, which was guided through the glass fiber, and a metal cap attached to the top of the glass fiber (arrow) was heated with the laser emission

Pyriform sinus fistula (PSF) is a rare congenital abnormality that causes acute suppurative thyroiditis [1]. PSF is an internal fistula arising from the apex of the pyriform recess and runs caudally anterior to the recurrent laryngeal nerve. The fistula often passes between the thyroid cartilage and the cricoid cartilage and runs into the upper portion of the thyroid lobe to form several branches there or it may end posterior to the lobe [2]. The presence of the fistula facilitates the spread of bacterial infection from the pharynx to the thyroid [3]. With respect to clinical management, complete surgical resection of the PSF has been widely accepted as a definitive treatment [2, 4]. However, it is difficult to identify the fistula within the severe fibrosis and adhesion caused by the previous inflammation. Thus, unsuccessful surgery and postoperative complications, such as vocal cord paralysis, may occur [4]. An endoscopic chemo-cauterization (ECC) treatment to obliterate the orifice of the fistula was devised by Kim et al. [5], and we adopted the technique in 2007 [6]. This procedure involves a short operation time, is minimally invasive, and has an acceptable complication rate. In addition, it is safe even for patients who have previously undergone open fistulectomy. Other methods to obliterate the fistula, like electrocauterization and CO_2_ laser-cauterization, have been developed [7, 8]. However, the success rate for fistula obliteration varies widely from 50 to 91%, and some cases require multiple procedures [5–10]. To improve the success rate, we adopted an endoscopic laser-cauterization (ELC) system, Diode Laser System ADL-20 (Asuka Medical Inc., Kyoto, Japan), in 2015. The Diode Laser System ADL-20 was previously used for endovascular laser photocoagulation therapy for the patients with chronic venous insufficiency, resulting in excellent clinical and safety outcomes [11]. The usual aim of laser treatments, such as CO_2_ lasers, is to irradiate lesions directly with a laser, but this comes with a risk of cauterizing not only the mucous membrane but also the deep tissues. However, the ELC system that we adopted selectively irradiates a metallic cap that is attached to the tip of the glass fiber with a laser (Fig. 1). We believe that this laser system can ablate the mucous membrane of the orifice portion of the fistula more accurately than ECC, making it a more suitable approach. Patients who were ineligible for the endoscopic procedures underwent conventional open fistulectomy. In this study, we aimed to evaluate the outcomes of these three treatment modalities.

## Materials and methods

The subjects were 83 patients with PSF who underwent treatment for fistula elimination between January 2007 and December 2018 at Kuma Hospital, which is a tertiary thyroid treatment hospital. Data were collected from the medical records. Imaging techniques used for diagnosis included ultrasonography, computed tomography (CT) scans with or without contrast enhancement, and barium swallow studies. Ultrasonography and CT imaging were used to evaluate the site and extent of the inflammation. CT was performed under a trumpet maneuver with the intention of using the air as a contrast agent to reveal the fistula [12]. Except for one patient, diagnoses were confirmed using barium swallow studies. The remaining patient presented with clinical features typical for PSF, but the results of the barium swallow study were inconclusive. The patient underwent suspension laryngoscopy for ECC, and the fistula was confirmed via endoscopy.

Of the 83 patients, 74 patients underwent endoscopic cauterization (ECC, 60 patients; ELC, 14 patients), whereas the remaining nine patients underwent open fistulectomy with or without partial hemithyroidectomy.

### Treatment protocol and follow-up

For the endoscopic procedures, the patient’s neck was flexed, and the head was placed in a suspended position under general anesthesia. Then, the suspension laryngoscope (Karl Storz GmbH & Co. KG, Germany) was inserted to access the pharynx. As the orifice of the PSF was usually located at the most caudal part of the pyriform recess, which was typically not fully open, we opened the recess with a silicon tube attached to the laryngoscope to obtain a clear view of the orifice. Then, the orifice of the PSF was identified with a rigid endoscope.

### Endoscopic chemo-cauterization

The ECC procedure is described in detail in our previous report [6]. Briefly, under the rigid endoscope, a small cotton ball soaked in 30% trichloroacetic acid was placed into the orifice for one minute, and the procedure was repeated 3–6 times until the cauterized membrane turned white. The membrane was then left to heal secondarily.

### Endoscopic laser-cauterization

ELC was performed using a Diode Laser System ADL-20 (Asuka Medical Inc., Kyoto, Japan) that generates 810-nm wavelength diode laser [11]. The diode laser is guided through a glass fiber. A metal cap attached to the top of the glass fiber is selectively heated with the laser during the active mode (Fig. 1). We thought that this system would be appropriate to cauterize the mucosa of the fistula selectively. The device has been approved by the Ministry of Health, Labor and Welfare and is covered by the healthcare insurance system in Japan to use for cutting, coagulating, and establishing hemostasis during surgical, otolaryngological [11], and bronchoscopic treatments. Prior to clinical use, we conducted a pre-clinical experiment using a raw chicken esophagus to test the amount of heat needed to cauterize the mucosal layer selectively, with minimal heat denaturing of the muscle layer (Fig. 1). We found that the optimal total dose was 360 J delivered intermittently at 20 s for 0.01 s at 6 J/s, repeated six times (unpublished data).

**Fig. 2:**
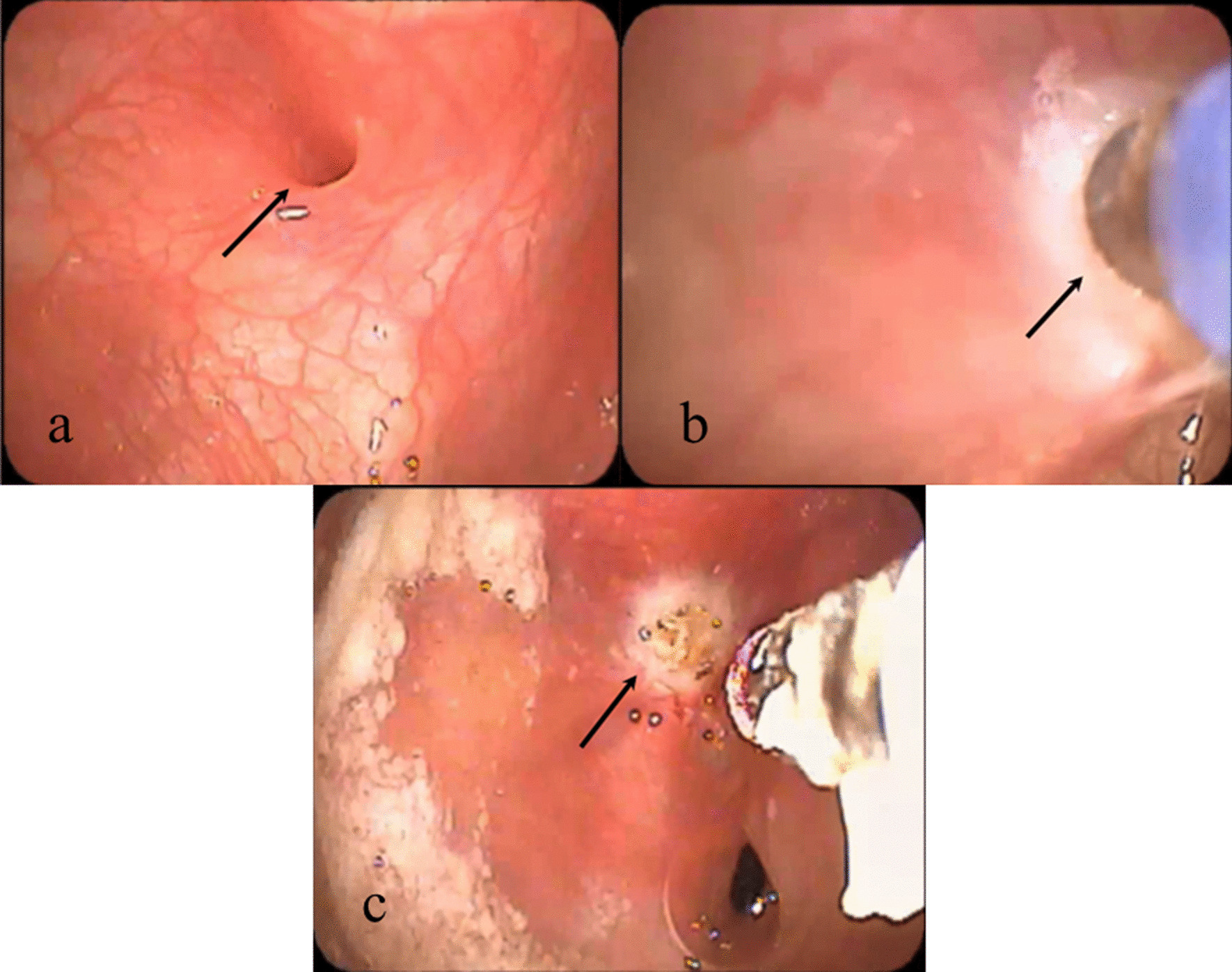
Endoscopic views of the left pyriform sinus fistula (PSF). Arrows show the orifice of the PSF. **a** The orifice of the PSF before treatment. **b** A metallic cap of the diode glass fiber tip was inserted into the PSF, and the mucous membrane being cauterized. **c** The orifice of the PSF turned white after the laser cauterization

After identification of the orifice of the PSF with a rigid endoscope (Fig. 2a), the operator replaced the endoscope to a flexible one. A metal-capped glass fiber was inserted through an instrument channel of the flexible endoscope into the PSF and was heated on active mode for 10 s (Fig. 2b). The procedure was repeated three times to coagulate for about 0.5 cm until the cauterized membrane turned white (Fig. 2c). The membrane was then left to heal secondarily. For precision, the operating mode was set to an intermittent pulse mode, alternately turning on and off every 0.01 s. The total energy delivery ranged from 60 to 300 J. Initially, air was sent to the metal cap through a channel within the glass fiber to avoid overheating during the procedure. However, this step was eliminated after we encountered a complication of subcutaneous emphysema in a patient as we describe below and considering that the use lasted for only 10 s.

All patients or their parents were informed that these endoscopic procedures were novel trials. They were also given the option of conversion to open fistulectomy when the endoscopic procedures were not possible. ECC and ELC for PSF treatments were approved by the institutional Ethical Committee at Kuma Hospital (20060713-1 and 20150709-2). All patients or their parents provided informed consent to undergo their preferred procedure.

### Open fistulectomy

Open fistulectomy was performed through a collar skin incision under general anesthesia using the pharyngeal approach technique reported by Nonomura et al. [13] in the four difficult cases.

### Postoperative course and follow-up

After the endoscopic procedures were carried out, patients received antibiotic treatment via intravenous infusion for five days to prevent local infection. The patients were provided with liquid diets one day after the procedures and solid diets four days after the procedures.

For open fistulectomy, patients received antibiotic treatment via intravenous infusion only during the operation. The patients were provided with solid diet one day after surgery.

The length of hospital stays for patients who underwent endoscopic procedures ranged from seven to eight days, which was the same as for patients who underwent open fistulectomy.

Posttreatment follow-up involved barium swallow studies and CT imaging under a trumpet maneuver to evaluate obliteration of the fistula at three months and one year after treatment. The patients were followed up for a median of 12 months (range, 1–144 months).

### Statistical analysis

The Mann–Whitney U test was used to compare variables. A Fisher’s exact test was used on 2 × 2 contingency tables. All statistical analyses were performed using StatFlex 6.0 software (Artech Co., Osaka, Japan). A *p* value < 0.05 was considered significant.

## Results

### Patient characteristics

The cohort comprised 51 female and 32 male patients. The median age at the first inflammatory episode was 10 years, and the median age at the first treatment at our institute was 16 years. The patients’ characteristics are shown in Table 1. The age at the first treatment at our institute was significantly higher in the open fistulectomy treatment group than that in the two endoscopic treatment groups. Thirty-eight patients had undergone incisional drainage at least once. Fourteen patients had previously undergone fistulectomy at other hospitals, but the treatments were unsuccessful. Except for the age at the first treatment at Kuma Hospital, the clinical features were not significantly different between the endoscopic treatment groups and the open fistulectomy group.

**Table 1:** Clinical features of patients with pyriform sinus fistula

	Overall cohort (n = 83)	Endoscopic treatment (n = 74)	Open fistulectomy (n = 9)	p value
Age at the first episode (years)^#^	10 (1–50)	8.5 (5–18)	15 (9.5–46)	n.s
Age at the first treatment at our institute (years)^#^	16 (4–62)	14.5 (7–24)	35 (15–54.5)	*p = 0.011
Sex: Female/male	32/51	29/45	3/6	n.s
Laterality				
Left	76 (91.6)	68 (91.9)	8 (88.9)	n.s
Right	6 (7.2)	5 (6.8)	1 (11.1)	
Bilateral	1 (1.2)	1 (1.4)	0 (0.0)	
Number of inflammation episodes				
Once	31 (37.3)	30 (40.5)	1 (11.1)	n.s
Twice	21 (25.3)	16 (21.6)	5 (55.6)	
≥ 3	31 (37.3)	28 (37.8)	3 (33.3)	
Clinical manifestations				
Abscess	47 (56.6)	44 (59.5)	3 (33.3)	n.s
Reddish swelling	34 (41.0)	29 (39.2)	5 (55.6)	
Simple mass	2 (2.4)	1 (1.4)	1 (11.1)	
History of incisional drainage				
None	45 (54.2)	40 (54.1)	5 (55.6)	
Once	25 (30.1)	23 (31.1)	2 (22.2)	n.s
≥ 2	13 (15.7)	11 (14.9)	2 (22.2)	
Previous fistulectomy	14 (16.9)	13 (17.6)	1 (11.1)	n.s

^#^ indicates median (ranges), other parentheses indicate %, n.s not significant

### Diagnostic and treatment outcomes

The results of barium swallow studies revealed a fistula in 82 (98.8%) of the 83 patients. CT imaging under a trumpet maneuver in 77 patients demonstrated a PSF in 31 patients (40.2%), and an air bubble in the thyroid lobe in six patients (7.8%) confirming the presence of a route from the pharynx to the thyroid. Although CT had a lower diagnostic rate for PSF than the barium swallow study, it demonstrated the anatomical path of the fistula more precisely.

Nine of the patients underwent open fistulectomy (Table 2). Two of these patients were treated before we adopted ECC treatment. Three patients were indicated for open surgery due to co-existing papillary thyroid carcinoma (n = 1), presence of an unusual fistula between the thyroid and an esophageal diverticulum (n = 1), and a long and wide funnel-shaped PSF regarded not suitable for endoscopic coagulation therapy (n = 1). In one patient, the orifice of PSF was not identified under laryngoscopy and thus the treatment was converted to open surgery. In the remaining three patients who were aged at least 35 years, the opening of their mouth and flexion of their neck were insufficient to insert the suspension laryngoscope. Of the nine patients treated with open fistulectomy, three patients underwent only fistulectomy, whereas the others underwent fistulectomy with hemithyroidectomy or partial hemithyroidectomy because the fistulae entered the thyroid lobe or because of co-existing papillary thyroid carcinoma.

**Table 2:** Indications for Surgery in the Open Fistulectomy Group (n = 9)

Case	Sex	Age (years)	Affected side	Indication of surgery	Operative procedure
1	Female	21	Left	Before introduction of endoscopic treatment	Lo + Fx
2	Male	62	Left	Before introduction of endoscopic treatment	Fx
3	Male	9	Left	Co-existing fistula between thyroid and esophageal diverticulum	Lo + Fx
4	Female	15	Left	Co-existing papillary thyroid carcinoma	Lo + Fx + CND
5	Male	17	Left	Large and long funnel-shaped fistula	Lo + Fx
6	Female	54	Left	Cannot detect the orifice of the PSF under laryngoscopy	Pl + Fx
7	Female	35	Left	Insufficient opening of the mouth	Lo + Fx
8	Female	46	Left	Insufficient opening of the mouth	Fx
9	Female	56	Right	Insufficient opening of the mouth	Fx

*CND* central neck dissection, *Fx* fistulectomy, *Lo* hemithyroidectomy, *Pl* partial hemithyroidectomy, *PSF* pyriform sinus fistula

### Obliteration of the fistula

Posttreatment barium swallow studies in 81 patients showed obliteration of the fistula following the first treatment at our institution in 70 (86%) patients. The success rates with respect to fistula obliteration in ECC, ELC, and open fistulectomy were 83% (49/59), 100% (13/13), and 100% (9/9), respectively. There were no significant differences between the groups (ECC vs ELC, p = 0.191; ECC vs open fistulectomy, p = 0.337; ELC vs open fistulectomy, p = 1.00). ECC was performed simultaneously with incisional drainage for a small neck abscess in two patients. The remaining two patients did not undergo postoperative evaluation studies. One patient in the ELC group refused barium swallow study but did not develop recurrence of the inflammation for more than one year after the treatment. Another patient was lost to follow-up.

**Fig. 3:**
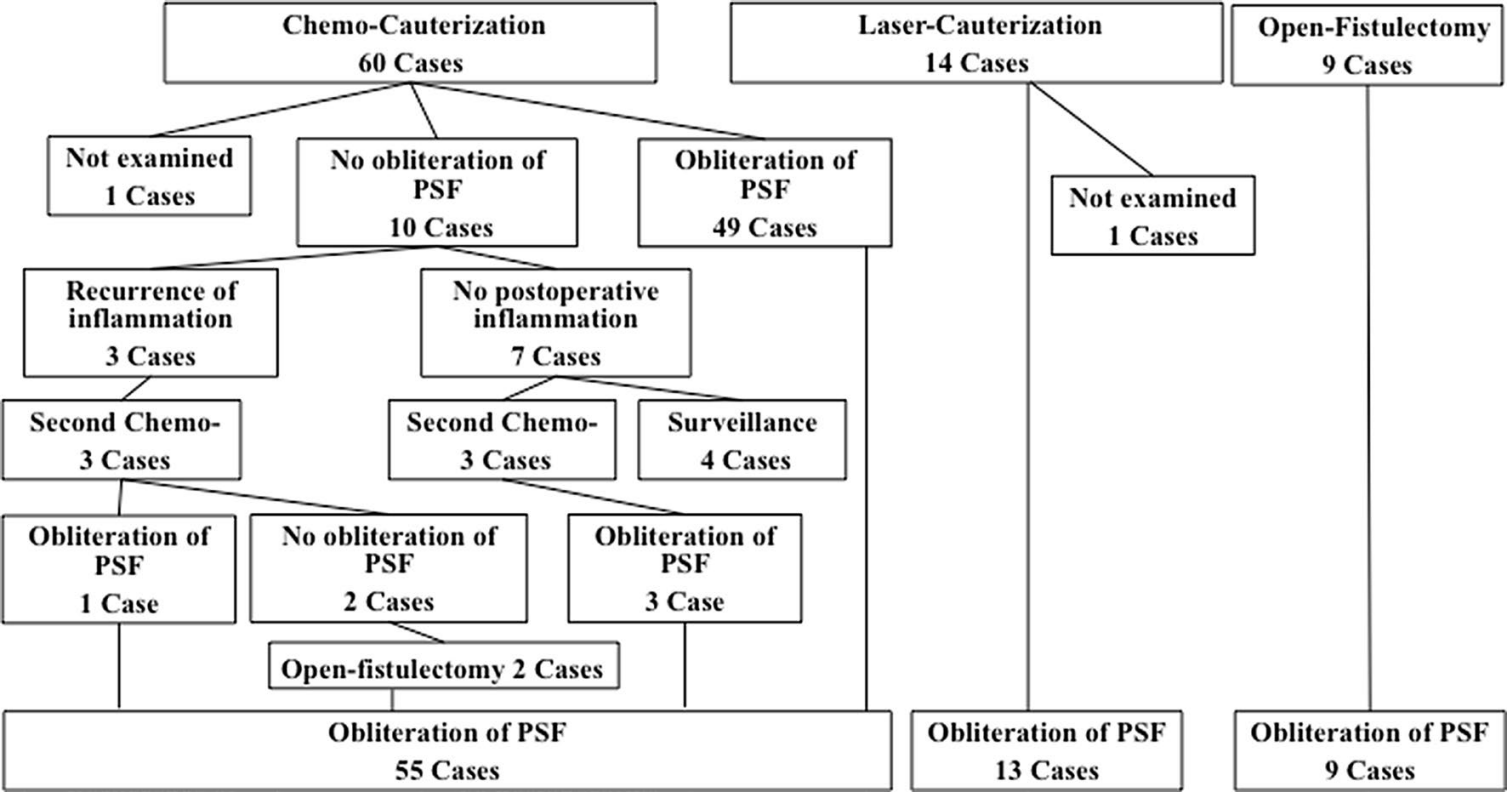
Flowchart for the outcomes of the three treatments

Figure 3 shows the treatments and outcomes. Elimination or obliteration of PSF was successfully achieved in all the patients in the open fistulectomy group and the ELC group, except in one patient who refused follow-up. Further, none of the patients experienced recurrence of the inflammation. Meanwhile, of the 60 patients in the ECC group, obliteration of the fistula was achieved in only 49 patients, and none of these patients developed recurrence of the inflammation. Obliteration was not achieved in the remaining 10 patients, three of whom experienced recurrence of the inflammation. These three patients underwent second ECC, but it was only successful in one patient. The remaining two patients required open fistulectomy. Meanwhile, the other seven of the 10 patients with a persistent fistula did not develop recurrence of the inflammation. However, three of them preferred second ECC, which achieved obliteration of the fistula, whereas the remaining four patients preferred surveillance without additional intervention. Thus, 53 (88%) of the 60 patients treated with ECC achieved obliteration of the PSF, although six patients required second and third treatments.

### Operative outcomes and complications

ECC required significantly less operative time (28 min, median) than did open fistulectomy (90 min, median) (P < 0.001). ELC required significantly lower operative time (13 min, median) than did the other treatments (vs ECC, P = 0.005; vs open fistulectomy, P < 0.001) (Table 3). Blood loss was almost zero in both endoscopic procedures and was significantly lower than that in the open fistulectomy group (Table 3).

**Table 3:**
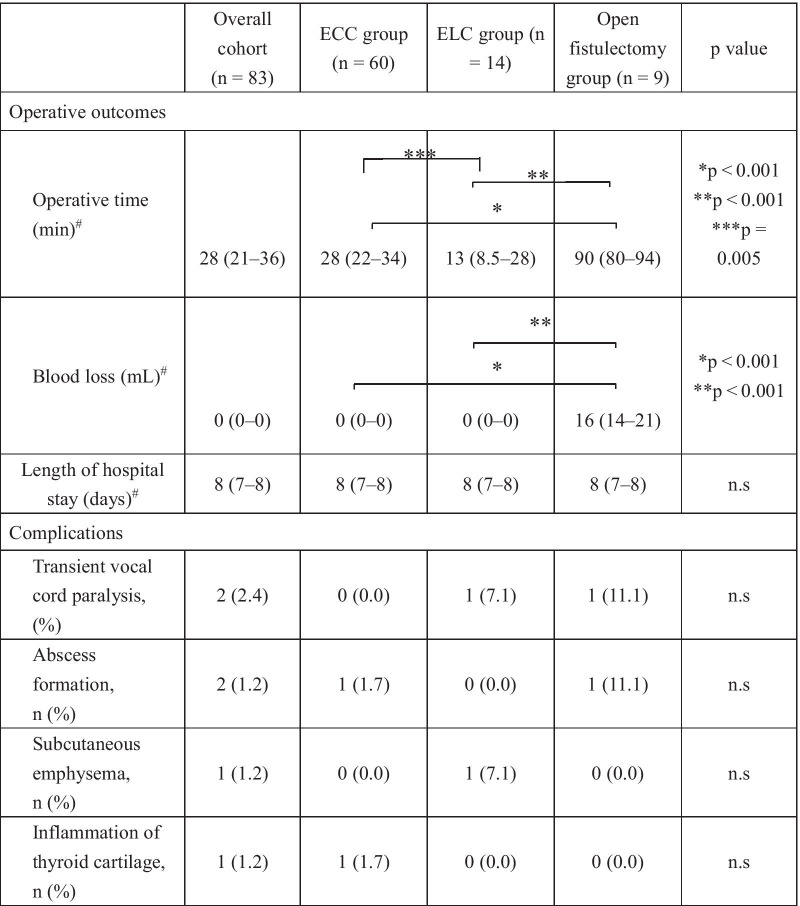
Operative outcomes and complications

^#^Median (range), other parentheses indicate %, *ECC* endoscopic chemo-cauterization, *ELC* endoscopic diode laser-cauterization, *n.s* not significant

None of the patients developed severe complications such as permanent vocal cord paralysis, dysphagia, and pharyngeal fistula (Table 3). However, transient vocal cord paralysis occurred in two patients after the treatments, but these were recovered within three months. Small abscess around the thyroid developed a few days after the treatments in two patients. The abscesses were adequately managed with incisional drainage and intravenous antibiotics. Patients that achieved obliteration of the fistula by ECC or ELC retained the caudal part of the PSF, but neither recurrence of inflammation nor cystic mass formation was observed.

One patient in the ELC group developed posttreatment subcutaneous emphysema that spread to the mediastinum forming a mild pneumothorax. The patient complained of mild chest pain without dyspnea, but this resolved spontaneously. Specifically, to avoid overheating of the cap and to ensure continuous use, the device was designed to send air to the metal cap through a channel in the glass fiber via a compressor. We believe that the air sent to the metal cap was the cause of the complication. Thus, we decided not to send air after this occurrence even though we used the device for only 10 s. This modification did not cause any problems thereafter. After ECC, one patient developed an unusual inflammatory change of the thyroid cartilage. She was referred to a university hospital and was treated with antibiotics. The inflammation disappeared two months after the treatment.

## Discussion

Although ECC is less invasive than open surgery, has a low risk of vocal cord paralysis, and is not associated with surgical scarring, the success rate for fistula obliteration using ECC was 83%, which requires improvement [6]. To improve obliteration of the fistula, we adopted a novel ELC system in 2015. In the current study, although the proportion of patients who underwent ELC was smaller than those who underwent ECC and open fistulectomy, the success rate for fistula obliteration was 100%, equivalent to that of open fistulectomy. The high obliteration rate for ELC treatment could be attributed to the higher precision and effectiveness of the laser ablation than that of chemo-cauterization. Specifically, a Diode Laser System ADL-20 generates 810-nm wavelength laser during the active mode, which is guided through a glass fiber. A metallic cap attached to the tip of the glass fiber is selectively heated with a laser; therefore, we could ablate more selectively and more completely the full layer of the mucous membrane of the orifice portion of the PSF as compared with ECC method. We believe that this system is preferable for fistula obliteration procedures. In addition, ELC also has the advantages of not requiring a surgical wound, short operating time, and lesser pain.

However, excess heat may cause thermal injury to the recurrent laryngeal nerve if it runs near the fistula. In the present study, one patient developed transient vocal cord paralysis after ELC treatment. It is difficult to estimate the running course of the recurrent laryngeal nerve in the pharynx, and thus the fistula should be treated carefully, particularly along the cricothyroid joint where the nerve should run close to the fistula. Jordan et al. [14] performed endoscopic obliteration of PSF with an insulated electrocautery probe, but the control of the spread of heat and electric effects might be more difficult in this technique than that in ELC technique.

Meanwhile, ELC also has several limitations. First, we need to insert a laser glass fiber into the fistula through a flexible laryngoscope. This may be difficult depending on the location of the orifice of PSF. Second, if the fistula has a large orifice or if it is funnel-shaped, the tip of the fiber may be too small to cauterize the fistula or we may need to insert the tip deeply to fit the size of the fistula. This might cause heat injury to the recurrent laryngeal nerve as described above. In such cases, ECC or open fistulectomy should be considered. Third, the suspension laryngoscope cannot be inserted in patients with insufficient opening of the mouth and flexion of the neck, particularly middle-aged or older patients. Fourth, this study is a retrospective study and a small number of patients were treated with the novel ELC method. Moreover, the high success rate of obliterating the fistula using ELC treatment may depend on the strict selection of the patients. Further studies are needed to evaluate endoscopic procedures for this rare internal fistula.

## Conclusions

Even though a small number of patients were treated, ELC achieved a higher obliteration rate of the fistula than did ECC treatment, and it achieved a similar rate to that achieved with open fistulectomy. Thus, ELC is potentially the optimal treatment modality for PSF. However, ECC and open fistulectomy are still important modalities for patients with a large opening, with a large and long funnel-shaped fistula, or with other lesions.

## Data Availability

The datasets supporting the conclusions of this article are included within the article.

## Abbreviations

PSF: Pyriform sinus fistula
ECC: Endoscopic chemo-cauterization
ELC: Endoscopic diode laser-cauterization
CT: Computed tomography
